# Solid-state structure and supra­molecular packing of 1,2-bis­(2-phenyl-1*H*-benzo[*d*]imidazol-1-yl)diazene

**DOI:** 10.1107/S2056989026005098

**Published:** 2026-05-22

**Authors:** Christos P. Constantinides, Jin-Seok Yi, Simona Marincean

**Affiliations:** a Department of Natural Sciences, University of Michigan-Dearborn, Dearborn MI 48128, USA; Indian Institute of Science Education and Research Bhopal, India

**Keywords:** benzimidazole, azo bond, π–π stacking, N-rich heterocycle, energetic materials, crystal structure

## Abstract

The title benzimidazole derivative, C_26_H_18_N_6_, which contains two benzimidazole units connected through an azo (N=N) linkage, was isolated as yellow needle-shaped crystals and characterized by single-crystal X-ray diffraction. The compound crystallizes in the monoclinic crystal system (space group: *P*2_1_*/c*) with one mol­ecule in the asymmetric unit. The dominant inter­actions in the crystal are parallel π–π contacts, giving rise to one-dimensional π-stacks along the *a-*axis direction, with a centroid-to-centroid separation of 3.899 (1) Å, a slippage distance of 1.81 Å, and a slip angle of 27.61°. Within these stacks, two short C—H⋯C contacts of 2.74 Å are observed between adjacent mol­ecules, which may reflect packing constraints. Along the *bc* plane, the structure comprises two-dimensional ribbons formed by anti­parallel chains. No short inter­planar contacts are observed between neighboring stacks.

## Abstract 2

The title compound crystallizes in the monoclinic space group *P*2_1_/*c*. The primary inter­molecular inter­actions are π–π stacking with the mol­ecules organized in one-dimensional π-stacks along the *a*-axis direction and two-dimensional ribbons parallel to the *bc* plane.

## 1. Chemical context

Compounds with multiple nitro­gen-rich heterocycles have attracted significant inter­est in the design of energetic materials (Lv *et al.*, 2024 ▸). The desired characteristics of energetic materials are tailored detonation performance and safety in extreme environments, which frequently exhibit opposite trends. The presence of multiple nitro­gen atoms in the backbone, either within heterocycles or as diazene (azo, –N=N–) linkages connecting aromatic moieties, has been reported to improve detonation performance due to release of higher decomposition energy (Beharry *et al.*, 2011 ▸; Tamaoki, 2001 ▸; Ikeda & Tsutsumi, 1995 ▸). Moreover, the presence of a diazene linkage can confer higher stability when conjugated with aromatic rings while improving detonation power due to the added nitro­gen content (Izsák *et al.*, 2013 ▸; Klapötke *et al.*, 2012 ▸; Hervé *et al.*, 2010 ▸). The crystal packing is critical to the stability of these materials, as external effects can be mitigated by inter­layer sliding (Zhang *et al.*, 2008 ▸). Thus, hydrogen bonding and π–π stacking inter­actions facilitated by aromatic moieties and nitro­gen-based proton acceptors are important design considerations for these materials.

Aromatic diazene compounds have found applications in therapeutics, food science, and radical chemistry. They have also attracted the attention of researchers working on photochemical mol­ecular switches (Cisnetti *et al.*, 2004 ▸), liquid crystal materials (Bandara & Burdette, 2012 ▸; Ikeda & Tsutsumi, 1995 ▸; Tamaoki, 2001 ▸), biomedical imaging (Beharry *et al.*, 2011 ▸), and light-driven mol­ecular motors (Murakami *et al.*, 1997 ▸). In our group, we have been inter­ested in amidrazonyl-based Blatter radicals with applications in spintronics and magnetism (Constanti­nides & Koutentis, 2016 ▸; Constanti­nides *et al.*, 2014 ▸, 2015 ▸, 2016 ▸, 2017 ▸, 2020 ▸; Nicolaides *et al.*, 2023 ▸; Perras *et al.*, 2022 ▸, 2023 ▸; Zissimou *et al.*, 2016 ▸; Bazzi *et al.*, 2020 ▸; Boudalis *et al.*, 2023 ▸). In the present paper, we report the X-ray structure of 1,2-bis­(2-phenyl-1*H*-benzo[d]imidazol-1-yl)diazene, (**I**) (Pozharskii *et al.*, 1989 ▸). Compared to other diazene-based energetic materials, this compound is unique in that the N=N bond directly links the two imidazole ring nitro­gen atoms, which may influence both detonation energy and stability. This work is an integral part of our ongoing research into how noncovalent inter­actions and extended conjugation influence the spectroscopic and magnetic properties of nitro­gen-rich heterocyclic compounds and related radicals.
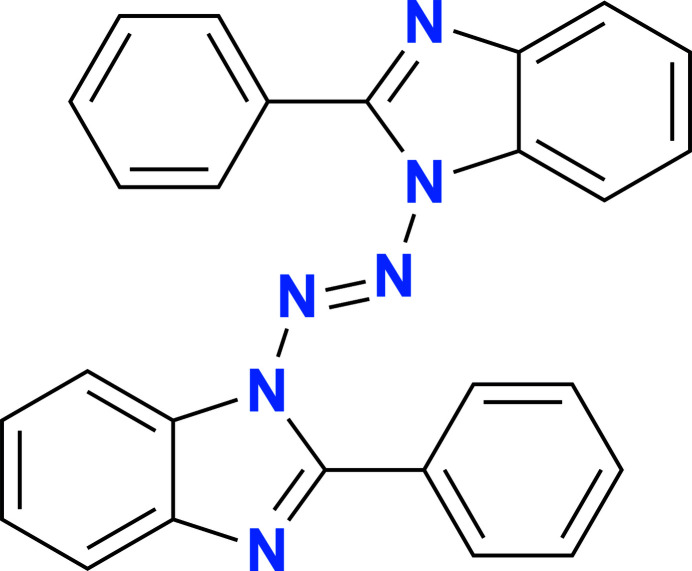


## 2. Structural commentary

**Figure 1:**
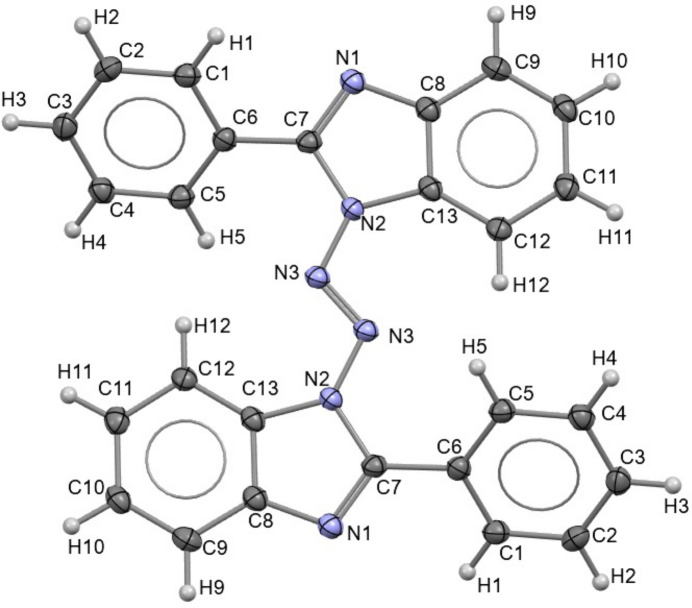
View of the mol­ecular structure of 1,2-bis­(2-phenyl-1*H*-benzo[*d*]imidazol-1-yl)diazene (**I**) (with atom numbering and ellipsoids drawn at the 50% probability level).

The title compound (Fig. 1 ▸) crystallizes in the monoclinic space group *P*2_1_/*c* with one mol­ecule in the asymmetric unit. The aromatic C—C bond lengths lie in the range 1.379 (2)–1.402 (2) Å, consistent with delocalized bonding in the aromatic rings. The phen­yl–benzimidazole connecting bond C6—C7 is 1.467 (2) Å, characteristic of a C*sp*^2^—C*sp*^2^ σ bond and indicative of limited electronic communication between the pendant phenyl rings and the benzimidazole core.

Within the imidazole ring, the angles around the N atoms (C7—N1—C8 and C7—N2—C13) are 105.4 (1) and 107.5 (1)°, respectively, while the remaining ring angles span 103.3 (1)–111.8 (1)°, with the largest value observed for N1—C7—N2 [11.9 (1)°], consistent with typical imidazole geometries (Cabildo *et al.*, 2015 ▸). In the fused benzene portion of the benzimidazole, the compressed angles at the fusion positions [*e.g.*, C8—C9—C10 ≃ 118.0 (1)° and C11—C12—C13 ≃ 116.6 (1)°] reflect the geometric constraints imposed by fusion to the imidazole ring, whereas the isolated phenyl rings exhibit angles close to 120° throughout.

**Figure 2:**
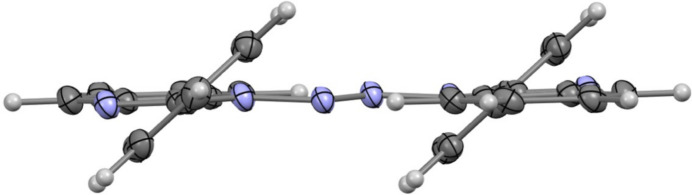
Side view of compound **I**, showing the dihedral angles of the phenyl rings (ellipsoids drawn at the 50% probability level).

A defining feature of the mol­ecule is the diazene (azo, –N=N–) linkage connecting the two benzimidazole units. The N—N distances N2—N3 and N3=N3 are 1.376 (1) and 1.259 (2) Å, respectively. The diazene adopts a *trans* (*E*) configuration, as shown by the N2—N3—N3—N2 torsion angle of −180.00 (9)°, placing the two benzimidazole fragments on opposite sides of the N=N bond. This arrangement is consistent with minimizing intra­molecular steric congestion in the N-rich core while maintaining a rigid, extended backbone. Accordingly, the benzimidazole–diazene core is close to planar, with only a small deviation of the benzimidazole unit relative to the diazene axis [*e.g*., C13—N2—N3—N3 = −6.7 (2)°]. In contrast, the two pendant phenyl rings are significantly rotated out of the core plane, with torsion angles of −40.6 (2) and −38.3 (2)° about the C6—C7 bond (Fig. 2 ▸). These twists further support reduced conjugation between the phenyl substituents and the benzimidazole–diazene core. The resulting geometry can therefore be described as a rigid, nearly planar diazene-linked benzimidazole scaffold bearing two twisted phenyl rings. Such a balance between core planarity and substituent rotation is expected to influence both the electronic structure and the packing preferences.

## 3. Supra­molecular features

**Figure 3:**
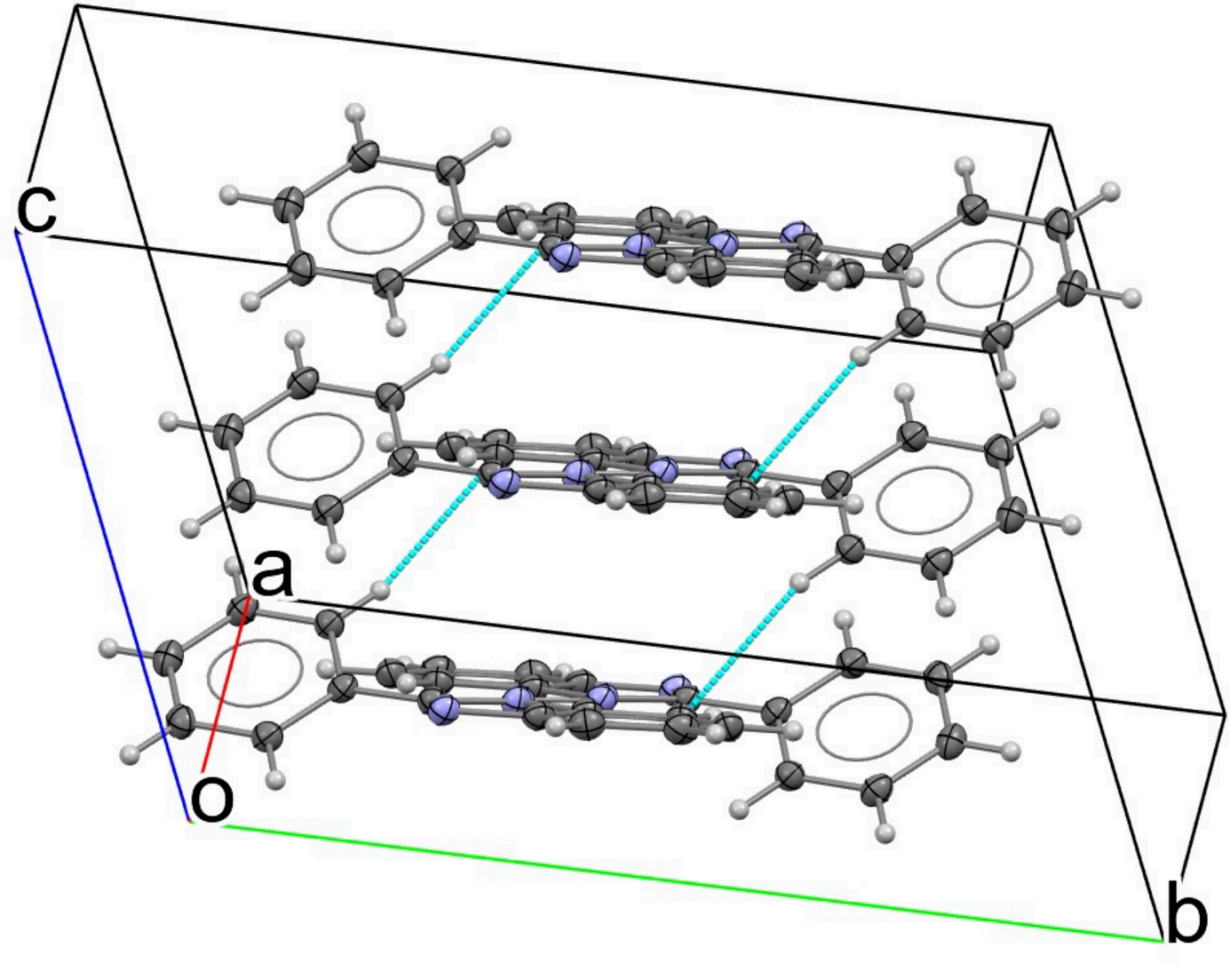
Packing along the *a* axis showing the one-dimensional slipped π-stack and intra­chain short C—H⋯C contacts (ellipsoids drawn at the 50% probability level).

The dominant packing motif is parallel-displaced π–π stacking between the diazene-linked benzimidazole cores. Along the *a-*axis direction, mol­ecules assemble into one-dimensional slipped π-stacks, with a centroid-to-centroid separation of 3.899 (1) Å, an interplanar separation of 3.454 (1) Å, and a slippage distance of 1.81 Å. Here, slippage is defined as the distance between one centroid and the perpendicular projection of the adjacent centroid onto the reference molecular plane. The corresponding slip angle, defined as the angle between the centroid-to-centroid vector and the plane normal, is 27.61° (Fig. 3 ▸). Within each stack, tight packing is further supported by a short C—H⋯C contact between neighboring mol­ecules (C5—H5⋯C12, H5⋯C12 = 2.74 Å, ∠C5—H5⋯C12 = 165.3°), which likely arises from the constrained geometry imposed by the close π-stacked arrangement.

**Figure 4:**
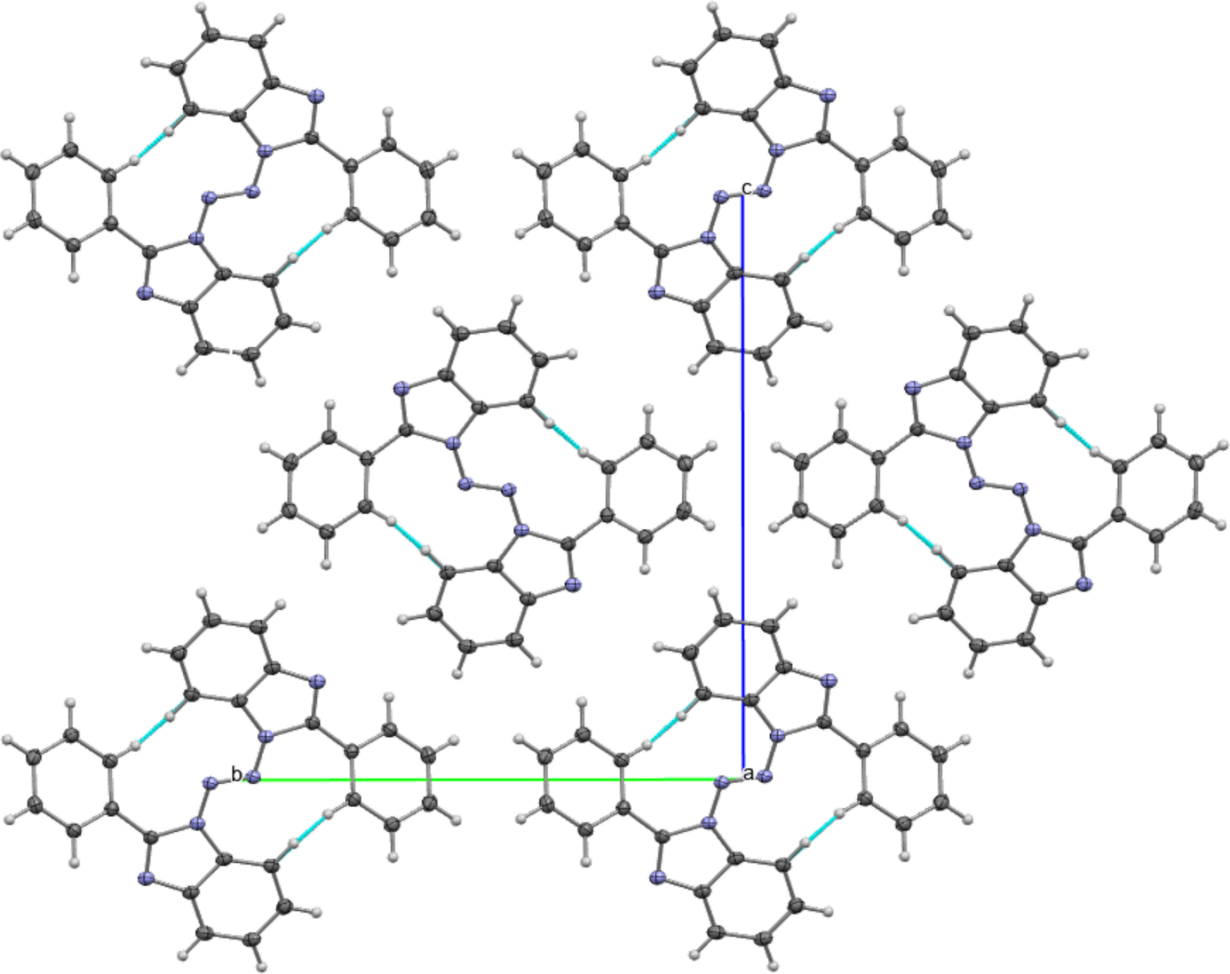
Two-dimensional ribbons along the *bc* plane Blue dotted lines indicate short intrachain C—H⋯C contacts between adjacent molecules. Ellipsoids are drawn at the 50% probability level.

Along the *b-*axis direction, the π-stacked columns pack in an anti­parallel fashion, giving rise to anti­parallel chains (Fig. 4 ▸). These chains align side-by-side to generate extended two-dimensional ribbons in the *bc* plane. In this arrangement, adjacent π-stacks are not linked by additional short inter­planar π–π contacts between neighboring columns, indicating that the association of the stacks into ribbons is governed primarily by close packing (dispersion-driven) contacts rather than face-to-face aromatic overlap.

## 4. Database survey

A search of Cambridge Structural Database (CSD, Version 6.01; update 2025.3, November 2025; Groom *et al.*, 2016 ▸) of moieties containing the tetra­zene (N—N=N—N) linker between two five-atom heterocycles led to 14 structures.

Those in the first group, *E*-1,2-bis­(3,5-di­nitro-1*H*-pyrazol-1-yl)diazene (COGDUZ; Yin *et al.*, 2014 ▸), 1,1′-diazene-1,2-diylbis(4,5-di­nitro-1*H*-imidazole) (COGFUB; Yin *et al.*, 2014 ▸), and 1,1′-diazene-1,2-diylbis(4,5-di­nitro-1*H*-imidazole) (COGGEM; Yin *et al.*, 2014 ▸) contain two imidazole rings functionalized with nitro groups. The rings and the diazo bond are planar with an extended conjugation, supported by the slight elongation of the diazo bond and compression of the adjacent N—N single bonds. The nitro groups have the O atoms out of the plane of the rings for COGDUZ and COGFUB. The supra­molecular arrangement exhibits parallel close contacts between the imidazole rings in the range 3.41–3.71 Å and close C—H⋯O contacts around 2.83 Å.

The remaining structures contain 4,4′-azo-1,2,4-triazole either functionalized at the rings or co-crystallized with hydrogen-bonding donors. The reference compound, 4,4′-azo-1,2,4-triazole, (ELAPOX; Qi *et al.*, 2011 ▸) exhibits an extended conjugation illustrated by the elongation of the N=N bond to 1.249 Å compared to 1.205 Å in tetra­zene (N_2_H_4_), and shortening of the adjacent N—N bonds to 1.371 compared to 1.429 Å in tetra­zene. The electron-withdrawing effect of the diazo group on the triazole leads to elongation of the C—N bonds, 1.373 and 1.369 Å, relative to the same bonds in 3-amino-1,2,4-triazole, 1.328 Å. Two inter­planar C—H_⋯_N hydrogen bonds are observed at 2.57 Å and 2.60 Å between two neighboring triazoles. The inter­planar distance of 3.257 (2) Å suggest π–π stacking inter­actions.

In the next set of compounds, the 4,4′-azo-1,2,4-triazole core was functionalized with an electron-withdrawing substituent [VETQOC: 3, 3′, 5, 5′ -N_3_ substituent; VETQES: 3,3′ -NP(Ph)_3_; VETQIW: 3,3′,5 -NP(Ph)_3_ substituent; Qi *et al.*, 2012 ▸] or electron-donating groups (VETQUI: 3, 3′, 5, 5′ NH_2_ substituent; Qi *et al.*, 2012 ▸). Two of the structures, VETQIW and VETQUI, crystallized with hydrogen-bonding solvents, CH_3_OH and H_2_O, respectively. The core structure is planar in all of these compounds with the exception of VETQUI where the triazole rings are slightly twisted out of plane, with a torsion angle of 168.3 (2)°. The degree of conjugation across the tetra­zene unit does not appear to depend strongly on whether the substituents are electron-donating or electron-withdrawing, as the relevant bond lengths remain essentially unchanged. Instead, the extent to which the substituents remain coplanar with the triazole rings is governed primarily by steric demands and the rigidity of the framework. N_3_ is in the plane of the triazole ring (VETQOC), but NH_2_ has only the N atom coplanar with the triazole, while the H atoms are twisted out of plane in VETQUI. In the presence of protic solvents in the crystal structures of VETQIW and VETQUI, a network of hydrogen bonds controls the supra­molecular arrangement. In absence of hydrogen bonding, the primary inter­molecular inter­actions are parallel π–π stacking at 3.010 (2) Å. Functionalization with bulkier substituents such as 3,3′-di­fluoro­azetidine does not impact the planarity of the central skeleton, leading to a reported increased π–π parallel stacking, 3.515 Å (GEWHOJ; Yang *et al.*, 2023 ▸).

Co-crystallization of 4,4′-azobis-1,2,4-triazole with acids, H_5_IO_6_ or HIO_3_, (AQEZOO and AQEZUU; Zhang *et al.*, 2021 ▸) led to structures characterized by O—H⋯N hydrogen bonds in the range 2.31–2.34 Å in a 3D network with inter­planar distances of 3.146 (12) and 3.231 (11) Å. Similarly, co-crystallization of 4,4′-azo-1,2,4-triazole with a series of polynitro­azoles (YOJXIH, YOJXON, YOKIFQ; Lu *et al.*, 2019 ▸) produced structures with stronger N—H⋯N hydrogen bonds between the triazole N atoms and the polynitro­azoles secondary amine moieties in the range 1.86–1.96 Å and additional C—H⋯H and C—H⋯O around 2.30–2.53 Å, significantly shorter than the close contacts in the structure of the unsubstituted 4,4′-azo-1,2,4 triazole, ELAPOX.

## 5. Refinement

Crystal data, data collection and structure refinement details are summarized in Table 1 ▸. H atoms were positioned geometrically and refined as riding [C—H = 0.95 Å, *U*_iso_(H) = 1.2*U*_eq_(C).].

## Supplementary Material

Crystal structure: contains datablock(s) I. DOI: 10.1107/S2056989026005098/dx2069sup1.cif

Structure factors: contains datablock(s) I. DOI: 10.1107/S2056989026005098/dx2069Isup2.hkl

Supporting information file. DOI: 10.1107/S2056989026005098/dx2069Isup3.cml

CCDC reference: 2544774

Additional supporting information:  crystallographic information; 3D view; checkCIF report

Additional supporting information:  crystallographic information; 3D view; checkCIF report

## Figures and Tables

**Table 1:** Experimental details

Crystal data	
Chemical formula	C_26_H_18_N_6_
*M*_r_	414.46
Crystal system, space group	Monoclinic, *P*2_1_/*c*
Temperature (K)	100
*a*, *b*, *c* (Å)	3.8987 (1), 14.7430 (4), 16.8695 (4)
β (°)	90.968 (2)
*V* (Å^3^)	969.50 (4)
*Z*	2
Radiation type	Cu *K*α
μ (mm^−1^)	0.70
Crystal size (mm)	0.14 × 0.09 × 0.04

Data collection	
Diffractometer	XtaLAB Synergy, Dualflex, HyPix-Arc 150
Absorption correction	Gaussian (*CrysAlis PRO*; Rigaku OD, 2025 ▸)
*T*_min_, *T*_max_	0.780, 1.000
No. of measured, independent and observed [*I* > 2σ(*I*)] reflections	6761, 1970, 1725
*R*_int_	0.025
(sin θ/λ)_max_ (Å^−1^)	0.628

Refinement	
*R*[*F*^2^ > 2σ(*F*^2^)], *wR*(*F*^2^), *S*	0.038, 0.109, 1.05
No. of reflections	1970
No. of parameters	145
H-atom treatment	H-atom parameters constrained
Δρ_max_, Δρ_min_ (e Å^−3^)	0.19, −0.23

Computer programs: *CrysAlis PRO* (Rigaku OD, 2025 ▸), *SHELXT2018/2* (Sheldrick, 2015*b* ▸), *SHELXL2019/3* (Sheldrick, 2015*a* ▸) and *OLEX2* (Dolomanov *et al.*, 2009 ▸).

## supplementary crystallographic information

### 1,2-Bis(2-phenyl-1H-benzo[d]imidazol-1-yl)diazene Crystal data

**Table:**

C_26_H_18_N_6_	*F*(000) = 432
*M**_r_* = 414.46	*D*_x_ = 1.420 Mg m^−^^3^
Monoclinic, *P*2_1_/*c*	Cu *K*α radiation, λ = 1.54184 Å
*a* = 3.8987 (1) Å	Cell parameters from 3254 reflections
*b* = 14.7430 (4) Å	θ = 4.0–75.2°
*c* = 16.8695 (4) Å	µ = 0.70 mm^−^^1^
β = 90.968 (2)°	*T* = 100 K
*V* = 969.50 (4) Å^3^	Needle, yellow
*Z* = 2	0.14 × 0.09 × 0.04 mm

### 1,2-Bis(2-phenyl-1H-benzo[d]imidazol-1-yl)diazene Data collection

**Table:**

XtaLAB Synergy, Dualflex, HyPix-Arc 150 diffractometer	1970 independent reflections
Radiation source: micro-focus sealed X-ray tube, PhotonJet (Cu) X-ray Source	1725 reflections with *I* > 2σ(*I*)
Mirror monochromator	*R*_int_ = 0.025
Detector resolution: 10.0000 pixels mm^-1^	θ_max_ = 75.5°, θ_min_ = 4.0°
ω scans	*h* = −4→4
Absorption correction: gaussian (CrysAlisPro; Rigaku OD, 2025)	*k* = −16→18
*T*_min_ = 0.780, *T*_max_ = 1.000	*l* = −20→17
6761 measured reflections	

### 1,2-Bis(2-phenyl-1H-benzo[d]imidazol-1-yl)diazene Refinement

**Table:**

Refinement on *F*^2^	Primary atom site location: dual
Least-squares matrix: full	Hydrogen site location: inferred from neighbouring sites
*R*[*F*^2^ > 2σ(*F*^2^)] = 0.038	H-atom parameters constrained
*wR*(*F*^2^) = 0.109	*w* = 1/[σ^2^(*F*_o_^2^) + (0.0681*P*)^2^ + 0.2069*P*] where *P* = (*F*_o_^2^ + 2*F*_c_^2^)/3
*S* = 1.05	(Δ/σ)_max_ < 0.001
1970 reflections	Δρ_max_ = 0.19 e Å^−^^3^
145 parameters	Δρ_min_ = −0.23 e Å^−^^3^
0 restraints	

### 1,2-Bis(2-phenyl-1H-benzo[d]imidazol-1-yl)diazene Special details

**Table:**

Geometry. All esds (except the esd in the dihedral angle between two l.s. planes) are estimated using the full covariance matrix. The cell esds are taken into account individually in the estimation of esds in distances, angles and torsion angles; correlations between esds in cell parameters are only used when they are defined by crystal symmetry. An approximate (isotropic) treatment of cell esds is used for estimating esds involving l.s. planes.
Refinement. The single-crystal X-Ray data were collected using a XtaLAB Synergy, Dualflex, HyPix-Arc 150 diffractometer operating at *T*= 100.00K. Data were measured using *w* scans with Cu K*_a_* radiation. The diffraction pattern was indexed and the total number of runs and images was based on the strategy calculation from the program CrysAlis^Pro^ 1.171.44.120a (Rigaku OD, 2025). The maximum resolution achieved was *Θ* = 75.492^°^ (0.80Å). The unit cell was refined using CrysAlis^Pro^ 1.171.44.120a on 3254 reflections, 48% of the observed reflections. Data reduction, scaling and absorption corrections were performed using CrysAlis^Pro^ 1.171.44.120a. The final completeness was 100 % out to 75.492^°^ in *Θ*. A gaussian absorption correction was performed using CrysAlis^Pro^ 1.171.44.120a. Numerical absorption correction based on gaussian integration over a multifaceted crystal model Empirical absorption correction using spherical harmonics, implemented in SCALE3 ABSPACK scaling algorithm.The structure was solved in the space group *P*2_1_/*c* by ShelXT 2018/2 (Sheldrick, 2015) using dual methods. It was refined by full matrix least squares minimisation on *F*^2^ using version 2019/3 of ShelXL 2019/3. All non-hydrogen atoms were refined anisotropically. Crystal data and details of the structure refinement for compound I are listed in Table 1.

### 1,2-Bis(2-phenyl-1H-benzo[d]imidazol-1-yl)diazene Fractional atomic coordinates and isotropic or equivalent isotropic displacement parameters (Å^2^)

**Table:**

	*x*	*y*	*z*	*U*_iso_*/*U*_eq_	
N2	0.6518 (3)	0.43294 (7)	0.42580 (6)	0.0190 (2)	
N3	0.4926 (3)	0.45780 (7)	0.49466 (5)	0.0191 (2)	
N1	0.8028 (3)	0.33259 (7)	0.33248 (6)	0.0214 (3)	
C13	0.7926 (3)	0.48524 (9)	0.36485 (6)	0.0195 (3)	
C5	0.6225 (3)	0.26215 (8)	0.53310 (7)	0.0210 (3)	
H5	0.731011	0.312099	0.558766	0.025*	
C6	0.5543 (3)	0.26544 (8)	0.45135 (7)	0.0197 (3)	
C7	0.6664 (3)	0.34175 (8)	0.40235 (7)	0.0194 (3)	
C8	0.8852 (3)	0.42032 (8)	0.30840 (7)	0.0205 (3)	
C4	0.5326 (3)	0.18647 (9)	0.57668 (7)	0.0231 (3)	
H4	0.578415	0.184831	0.632150	0.028*	
C12	0.8429 (3)	0.57754 (9)	0.35356 (7)	0.0208 (3)	
H12	0.773367	0.620998	0.391639	0.025*	
C1	0.3969 (3)	0.19115 (9)	0.41434 (7)	0.0218 (3)	
H1	0.350462	0.192463	0.358893	0.026*	
C10	1.1014 (3)	0.53880 (9)	0.22755 (7)	0.0239 (3)	
H10	1.211508	0.558540	0.180796	0.029*	
C2	0.3087 (3)	0.11579 (8)	0.45822 (7)	0.0235 (3)	
H2	0.201603	0.065510	0.432748	0.028*	
C9	1.0445 (3)	0.44739 (9)	0.23875 (7)	0.0238 (3)	
H9	1.111603	0.404152	0.200248	0.029*	
C3	0.3758 (3)	0.11306 (9)	0.53961 (8)	0.0244 (3)	
H3	0.314488	0.061131	0.569547	0.029*	
C11	1.0005 (3)	0.60313 (9)	0.28357 (7)	0.0232 (3)	
H11	1.040383	0.665637	0.273602	0.028*	

### 1,2-Bis(2-phenyl-1H-benzo[d]imidazol-1-yl)diazene Atomic displacement parameters (Å^2^)

**Table:**

	*U*^11^	*U*^22^	*U*^33^	*U*^12^	*U*^13^	*U*^23^
N2	0.0248 (6)	0.0184 (5)	0.0137 (5)	0.0002 (4)	0.0000 (4)	0.0000 (4)
N3	0.0238 (5)	0.0192 (5)	0.0144 (5)	0.0014 (4)	−0.0008 (4)	−0.0016 (4)
N1	0.0259 (6)	0.0215 (5)	0.0166 (5)	0.0012 (4)	−0.0010 (4)	−0.0002 (4)
C13	0.0203 (6)	0.0229 (7)	0.0151 (6)	0.0000 (4)	−0.0024 (4)	0.0013 (4)
C5	0.0242 (6)	0.0196 (6)	0.0191 (6)	0.0010 (5)	−0.0018 (4)	−0.0019 (4)
C6	0.0220 (6)	0.0183 (6)	0.0188 (6)	0.0030 (4)	0.0003 (4)	0.0004 (4)
C7	0.0231 (6)	0.0184 (6)	0.0167 (6)	0.0016 (4)	−0.0025 (4)	−0.0015 (4)
C8	0.0225 (6)	0.0224 (6)	0.0166 (6)	0.0006 (5)	−0.0027 (4)	0.0009 (4)
C4	0.0271 (6)	0.0232 (6)	0.0190 (6)	0.0038 (5)	0.0002 (4)	0.0017 (5)
C12	0.0233 (6)	0.0216 (6)	0.0175 (6)	−0.0013 (5)	−0.0025 (4)	−0.0004 (5)
C1	0.0253 (6)	0.0210 (6)	0.0189 (6)	0.0026 (5)	−0.0013 (4)	−0.0024 (5)
C10	0.0255 (6)	0.0301 (7)	0.0160 (6)	−0.0021 (5)	−0.0004 (5)	0.0025 (5)
C2	0.0258 (6)	0.0179 (6)	0.0269 (6)	0.0005 (5)	0.0005 (5)	−0.0037 (5)
C9	0.0258 (7)	0.0288 (7)	0.0168 (6)	0.0015 (5)	0.0002 (5)	−0.0016 (5)
C3	0.0274 (7)	0.0202 (6)	0.0256 (6)	0.0013 (5)	0.0047 (5)	0.0033 (5)
C11	0.0259 (7)	0.0231 (6)	0.0204 (6)	−0.0037 (5)	−0.0040 (5)	0.0029 (5)

### 1,2-Bis(2-phenyl-1H-benzo[d]imidazol-1-yl)diazene Geometric parameters (Å, º)

**Table:**

N2—N3	1.3760 (13)	C4—H4	0.9500
N2—C13	1.4044 (15)	C4—C3	1.3868 (18)
N2—C7	1.4028 (15)	C12—H12	0.9500
N3—N3^i^	1.259 (2)	C12—C11	1.3923 (17)
N1—C7	1.3081 (16)	C1—H1	0.9500
N1—C8	1.3949 (16)	C1—C2	1.3816 (18)
C13—C8	1.4018 (17)	C10—H10	0.9500
C13—C12	1.3885 (17)	C10—C9	1.3794 (19)
C5—H5	0.9500	C10—C11	1.4000 (18)
C5—C6	1.4010 (16)	C2—H2	0.9500
C5—C4	1.3845 (18)	C2—C3	1.3940 (18)
C6—C7	1.4672 (16)	C9—H9	0.9500
C6—C1	1.3974 (17)	C3—H3	0.9500
C8—C9	1.3963 (17)	C11—H11	0.9500
			
N3—N2—C13	131.25 (10)	C3—C4—H4	119.9
N3—N2—C7	120.96 (10)	C13—C12—H12	121.7
C7—N2—C13	107.54 (10)	C13—C12—C11	116.57 (12)
N3^i^—N3—N2	111.34 (12)	C11—C12—H12	121.7
C7—N1—C8	105.41 (10)	C6—C1—H1	119.9
C8—C13—N2	103.35 (11)	C2—C1—C6	120.15 (11)
C12—C13—N2	134.14 (11)	C2—C1—H1	119.9
C12—C13—C8	122.51 (11)	C9—C10—H10	119.3
C6—C5—H5	119.8	C9—C10—C11	121.45 (11)
C4—C5—H5	119.8	C11—C10—H10	119.3
C4—C5—C6	120.33 (11)	C1—C2—H2	119.8
C5—C6—C7	121.85 (11)	C1—C2—C3	120.44 (12)
C1—C6—C5	119.16 (11)	C3—C2—H2	119.8
C1—C6—C7	118.83 (10)	C8—C9—H9	121.0
N2—C7—C6	124.20 (10)	C10—C9—C8	118.00 (11)
N1—C7—N2	111.85 (10)	C10—C9—H9	121.0
N1—C7—C6	123.91 (10)	C4—C3—C2	119.71 (11)
N1—C8—C13	111.84 (10)	C4—C3—H3	120.1
N1—C8—C9	128.17 (11)	C2—C3—H3	120.1
C9—C8—C13	119.98 (12)	C12—C11—C10	121.46 (12)
C5—C4—H4	119.9	C12—C11—H11	119.3
C5—C4—C3	120.21 (11)	C10—C11—H11	119.3
			
N2—C13—C8—N1	0.70 (13)	C7—N2—N3—N3^i^	179.83 (12)
N2—C13—C8—C9	−178.46 (11)	C7—N2—C13—C8	−0.37 (12)
N2—C13—C12—C11	179.03 (12)	C7—N2—C13—C12	178.98 (13)
N3—N2—C13—C8	−174.54 (12)	C7—N1—C8—C13	−0.76 (14)
N3—N2—C13—C12	4.8 (2)	C7—N1—C8—C9	178.31 (12)
N3—N2—C7—N1	174.81 (10)	C7—C6—C1—C2	175.79 (11)
N3—N2—C7—C6	−7.29 (18)	C8—N1—C7—N2	0.50 (14)
N1—C8—C9—C10	−179.86 (12)	C8—N1—C7—C6	−177.41 (11)
C13—N2—N3—N3^i^	−6.66 (19)	C8—C13—C12—C11	−1.72 (18)
C13—N2—C7—N1	−0.08 (14)	C4—C5—C6—C7	−175.80 (11)
C13—N2—C7—C6	177.83 (11)	C4—C5—C6—C1	−0.49 (18)
C13—C8—C9—C10	−0.85 (18)	C12—C13—C8—N1	−178.75 (11)
C13—C12—C11—C10	0.20 (18)	C12—C13—C8—C9	2.09 (18)
C5—C6—C7—N2	−40.64 (18)	C1—C6—C7—N2	144.03 (12)
C5—C6—C7—N1	137.02 (13)	C1—C6—C7—N1	−38.31 (18)
C5—C6—C1—C2	0.34 (18)	C1—C2—C3—C4	−0.08 (19)
C5—C4—C3—C2	−0.07 (19)	C9—C10—C11—C12	0.97 (19)
C6—C5—C4—C3	0.36 (19)	C11—C10—C9—C8	−0.63 (19)
C6—C1—C2—C3	−0.06 (19)		

Symmetry code: (i) −*x*+1, −*y*+1, −*z*+1.

### Bond lengths (Å) in 1,2-bis(2-phenyl-1H-benzo[d]imidazol-1-yl)diazene (I)

**Table:**

Atom A	Atom B	A-B Bond Length (Å)
C13	N2	1.404 (2)
N2	C7	1.403 (2)
C7	N1	1.308 (2)
N1	C8	1.395 (2)
C8	C13	1.402 (2)
C13	C12	1.388 (2)
C12	C11	1.392 (2)
C11	C10	1.400 (2)
C10	C9	1.379 (2)
C9	C8	1.396 (2)
C7	C6	1.467 (2)
C6	C5	1.401 (2)
C5	C4	1.385 (2)
C4	C3	1.387 (2)
C3	C2	1.394 (2)
C2	C1	1.382 (2)
C1	C6	1.398 (2)
N2	N3	1.376 (1)
N3	N3'	1.258 (1)

### Bond angles (°) in 1,2-bis(2-phenyl-1H-benzo[d]imidazol-1-yl)diazene (I)

**Table:**

Atom A	Atom B	Atom C	A-B-C Bond Angle (°)
			
C13	N2	C7	107.5 (1)
N2	C7	N1	111.8 (1)
C7	N1	C8	105.4 (1)
N1	C8	C13	111.8 (1)
C8	C13	N2	103.3 (1)
C13	C12	C11	116.6 (1)
C11	C10	C9	121.5 (1)
C12	C11	C10	121.5 (1)
C10	C9	C8	118.0 (1)
C9	C8	C13	120.0 (1)
C8	C13	C12	122.5 (1)
N2	C7	C6	124.2 (1)
N1	C7	C6	123.9 (1)
N3	N2	C7	121.0 (1)
N3	N2	C13	131.2 (1)
N3	N3	N2	111.3 (1)
C6	C5	C4	120.3 (1)
C5	C4	C3	120.2 (1)
C4	C3	C2	119.7 (1)
C3	C2	C1	120.4 (1)
C2	C1	C6	120.1 (1)
C1	C6	C5	119.2 (1)

### Torsion angles (°) in 1,2-bis(2-phenyl-1H-benzo[d]imidazol-1-yl)diazene (I)

**Table:**

Atom A	Atom B	Atom C	Atom D	A-B-C-D
N2	C7	C6	C5	-40.6 (2)
N1	C7	C6	C1	-38.3 (2)
C13	N2	N3	N3	-6.6 (2)
N2	N3	N3	N2	-180.00 (9)
